# Exploring constructs of well-being, happiness and quality of life

**DOI:** 10.7717/peerj.4903

**Published:** 2018-06-01

**Authors:** Oleg N. Medvedev, C. Erik Landhuis

**Affiliations:** 1 School of Medicine, University of Auckland, Auckland, New Zealand; 2 School of Social Sciences and Public Policy, Auckland University of Technology, Auckland, New Zealand

**Keywords:** Quality of life, Well-being, Happiness, Measurement, Life satisfaction

## Abstract

**Background:**

Existing definitions of happiness, subjective well-being, and quality of life suggest conceptual overlap between these constructs. This study explored the relationship between these well-being constructs by applying widely used measures with satisfactory psychometric properties.

**Materials and Methods:**

University students (*n* = 180) completed widely used well-being measures including the Oxford Happiness Questionnaire (OHQ), the World Health Organization Quality of Life Questionnaire, the Satisfaction with Life Scale, and the Positive and Negative Affect Scale. We analyzed the data using correlation, regression, and exploratory factor analysis.

**Results:**

All included well-being measures demonstrated high loadings on the global well-being construct that explains about 80% of the variance in the OHQ, the psychological domain of Quality of Life and subjective well-being. The results show high positive correlations between happiness, psychological and health domains of quality of life, life satisfaction, and positive affect. Social and environmental domains of quality of life were poor predictors of happiness and subjective well-being after controlling for psychological quality of life.

**Conclusion:**

Together, these data provide support for a global well-being dimension and interchangeable use of terms happiness, subjective well-being, and psychological quality of life with the current sample and measures. Further investigation with larger heterogeneous samples and other well-being measures is warranted.

## Introduction

The existing definitions of happiness, subjective well-being, and health related quality of life and the main components assigned to these constructs in the research literature (see Table 1) suggest conceptual overlap between these dimensions (Camfield & Skevington, 2008). Quality of life was defined in the cross-cultural project of the World Health Organization (WHO) as:

An individual’s perception of their position in life, in the context of the culture and value systems in which they live, and in relation to their goals, expectations, standards, and concerns. It is a broad ranging concept, affected in a complex way by the person’s physical health, psychological state, level of independence, social relationships and their relationships to salient features of their environment.

**Table 1:** Components of happiness related constructs based on the research literature.

Components:	Affective		Cognitive			Physical
Constructs	Positive affect	Negative affect	Life satisfaction	Happy traits	Positive functioning	Perceived health
**Happiness**	Hills & Argyle (2002)	Hills & Argyle (2002)	Hills & Argyle (2002)	Hills & Argyle (2002)	Joseph & Lewis (1998)	Hills & Argyle (2002)
	Diener (1984)	Diener (1984)	Diener (1984)	Argyle (2001)	Ryff (1989)	Joseph & Lewis (1998)
	Joseph & Lewis (1998)	Joseph & Lewis (1998)	Joseph & Lewis (1998)	Fordyce (1988)		Eid & Larsen (2008)
	Eid & Larsen (2008)	Eid & Larsen (2008)	Eid & Larsen (2008)	Eid & Larsen (2008)		
	Brandburn (1969)	Brandburn (1969)				
	Fordyce (1988)	Fordyce (1988)				
**Subjective well-being**	Andrews & Withey (1976)	Andrews & Withey (1976)	Andrews & Withey (1976)	Diener (2006)	Diener (2006)	Diener (2006)
	Diener (1984)	Diener (1984)	Diener (1984)	Eid & Larsen (2008)		Eid & Larsen (2008)
	Diener (2006)	Diener (2006)	Diener (2006)	Hills & Argyle (2002)		Hills & Argyle (2002)
	Eid & Larsen (2008)	Eid & Larsen (2008)	Eid & Larsen (2008)			
	Hills & Argyle (2002)	Hills & Argyle (2002)	Hills & Argyle (2002)			
**Quality of life**	Andrews & Withey (1976)	Andrews & Withey (1976)	Andrews & Withey (1976)		WHOQOL Group (1998a, 1998b)	WHOQOL Group (1998a, 1998b)
	WHOQOL Group (1998a, 1998b)	WHOQOL Group (1998a, 1998b)	WHOQOL Group (1998a, 1998b)			

(WHOQOL Group, 1995, p. 1404)

The new reconceptualization of subjective well-being assumed to be synonymous of happiness by Diener (2006, p. 400) as: “An umbrella term for different valuations that people make regarding their lives, the events happening to them, their bodies and minds, and the circumstances in which they live” resulted in greater theoretical convergence between these constructs. This raises an issue as to the point in which conceptual overlap invites redundancy, and whether one or the other of the terms is now surplus to requirements.

Historically, humans strived to achieve happiness and considered it the most important goal in life (Compton, 2005). Cross-cultural research provide supporting evidence for primacy of happiness compared to other individual values such as physical health, wealth or love (Kim-Prieto et al., 2005; Skevington, MacArthur & Somerset, 1997). Essentially, other human goals are valued because they are believed to give rise to happiness (Csikszentmihaliy, 1992). Initially psychology was dealing with mental health issues affecting physical and social functioning of an individual (Andrews & McKennell, 1980; Beck, 1991, 1993). Happiness, well-being, and quality of life have only attracted increased interest of psychologists by the end of the 20th century resulting in growing research in this area (Diener, 1984; WHOQOL Group, 1998a, 1998b). Happiness and well-being research became increasingly important in the economics’ context (Kristoffersen, 2010), and well-being data are widely used along with economic indicators by economists (Kahneman & Krueger, 2006).

Currently, there is no agreement between researchers in defining happiness and its related constructs (Diener, 2006; Diener et al., 2010; Rojas & Veenhoven, 2013; Kern et al., 2014; Shin & Johnson, 1978). In the literature happiness is often called subjective well-being (Diener, 2006; Hills & Argyle, 2002), emotional well-being, positive affect (Brandburn, 1969; Fordyce, 1988), and quality of life (Diener, 2000; Ratzlaff et al., 2000; Shin & Johnson, 1978), which suggests that the meanings of happiness may depend on the context (Diener, 2006; Carlquist et al., 2016). Elsewhere, subjective happiness was defined as “a global evaluation of life satisfaction” (Diener, 2006, p. 400). In the same way, subjective well-being was defined as “evaluations of life quality” (Andrews & McKennell, 1980, p. 131). These definitions indicate close relationship between the constructs of happiness, subjective well-being, quality of life, and life satisfaction. More recently subjective well-being was proposed as more appropriate “Big One” including the relevant aspects of global well-being (Diener, 2006; Kashdan, Biswas-Diener & King, 2008).

Happiness can be described by bottom-up and top-down processes (Andrews & McKennell, 1980; Diener, 1984). The bottom-up approach implies that happiness depends on aggregated positive and negative feelings (Diener, 1984). However, evidence suggests that positive affect is not a counterpart of negative affect and the correlation between them is merely moderate (Argyle, 2001; Tellegen et al., 1988). Alternatively, top-down approaches explain happiness is a result of subjective evaluations of individual’s life experiences or satisfaction with life (SL) (Andrews & McKennell, 1980; Diener, Lucas & Oishi, 2005). The top-down approach has both theoretical foundation (Beck, 1993; Diener, 1984) and empirical support (Andrews & McKennell, 1980; Butler et al., 2006). The approaches appear to complement each other because research on happiness assessment consistently indicates that positive and negative affect and a cognitive component or SL map on unidimensional happiness construct (Argyle, 2001; Hills & Argyle, 1998, 2002; Joseph & Lewis, 1998). The cognitive component of happiness may involve personality traits such as optimism, extraversion, and internal locus of control (Fordyce, 1988; Mayers, 1992).

Shin & Johnson (1978) noted, “happiness has been mistakenly identified with feelings of pleasure” in the research literature and defined as emotional well-being (Fordyce, 1986; Layard, 2005). Shin & Johnson (1978) proposed that this definition refers to hedonic happiness associated with feeling happy also called euphoria or elation. They argued that *feeling happy* and *being happy* are not the same—*being happy* refers to enduring condition rather than to momentary pleasures or happy feelings. Accordingly, happiness should be understood as global evaluation of individual’s life quality according to their own criteria, which include both cognitions and emotions (Shin & Johnson, 1978). According to this model *feeling happy* refers to *state* happiness and *being happy* incorporates both *state* and *trait* happiness.

The hedonic concept of happiness does not consider that cognitive appraisal plays the important role in emotional functioning (Frijda, 1998, 2007). According to the dual route model of emotional processing proposed by LeDoux (2000), triggering information is simultaneously sent to the amygdala, resulting in immediate physiological responses like “fight or flight” (Cannon, 1929), and to prefrontal cortex for further cognitive appraisal. Evidence shows that activation of the amygdala could be inhibited by prefrontal brain structures involved in conscious cognition (Thayer et al., 2009; Thayer & Lane, 2000). Also, the impact of cognition on emotional states is well supported by evidence-based cognitive therapy (Butler et al., 2006; Ellis, 2002). Therefore, the definition of happiness as merely emotional well-being is limited, because it does not account for the cognitive component of happiness supported by both theories and empirical evidence (Diener et al., 1999; Eid & Larsen, 2008; Frijda, 2007).

Rather than constructing happiness as merely emotional well-being, Ryff (1989) and Ryff & Keyes (1995) proposed a eudemonic model of happiness, which they also called psychological well-being or positive functioning, comprising six dimensions: purpose in life; personal growth; environmental mastery; autonomy; positive self-regard; and social connections. These dimensions do not include basic components of subjective well-being and happiness such as emotions and life satisfaction consistently supported by the literature (Helliwell, Huang & Wang, 2014; Diener, Sapyta & Suh, 1998; Rojas & Veenhoven, 2013). The construct validity of the assessment instrument based on these six factors (Ryff, 1989) was challenged by later investigation indicating substantial overlap between dimensions (Springer & Hauser, 2006; Springer, Hauser & Freese, 2006).

Ryff’s (1989) model of eudemonic happiness was also scrutinized by Diener et al. (2010), resulting in development of an alternative construct defined as “psychological flourishing” or an individual’s self-perceived success, which is an aspect of life satisfaction. The proposed construct emphasizes positive functioning and covers dimensions such as social relationships; purposeful life; engagement in activities; self-esteem; and optimism, which overlap with components of widely used quality of life and happiness measures (WHOQOL Group, 1998a; Hills & Argyle, 2002). For instance, social relationships is a domain of the quality of life measure (WHOQOL Group, 1998a) and self-esteem and optimism are components of the widely used happiness measure (Hills & Argyle, 2002). The component “purposeful life” implies that one cannot be happy without having a purpose making happiness an exclusive attribute of a group of adults who managed to develop such a purpose. Including this component in a psychometric measure may violate fundamental measurement principle of invariance across population groups (Thurstone, 1931) because the sense of purpose in life varies substantially across cultural and age groups (Oishi & Diener, 2014). Notwithstanding the importance of eudemonic well-being associated with individual’s fulfilment, it is implicitly included in subjective well-being and reflected by the overall SL (Diener et al., 1999; Eid & Larsen, 2008; Kashdan, Biswas-Diener & King, 2008).

Different measures were developed to assess well-being associated constructs, however, definitions used in these instruments appear inconsistent (Diener et al., 1999; Diener et al., 2010; Fordyce, 1986; Joseph & Lewis, 1998). Also, one or two items often used to measure well-being or happiness in national and cross-cultural surveys appeared unreliable compared to measures with more items covering various well-being components (Andrews & McKennell, 1980; Hills & Argyle, 2002; Joseph & Lewis, 1998).

Hills & Argyle (2002) considered limitations of earlier happiness measurements when developing their Oxford Happiness Questionnaire (OHQ). The authors used the terms “well-being” and “subjective well-being” as synonyms for “happiness” when describing the OHQ. This instrument is a new version of the Oxford Happiness Inventory (Argyle, 2001) and both scales were widely used in Oxford University for assessment of personal happiness and are shown to have satisfactory psychometric properties (Hills & Argyle, 2002). The OHQ is a unidimensional scale that contains items tapping into positive and negative affect, life satisfaction and happy traits such as sense of control, physical fitness, positive cognition, mental alertness, self-esteem, cheerfulness, optimism, and empathy (Diener, 1984; Hills & Argyle, 2002).

Quality of Life was widely recognized as a health related issue associated with the WHO’s definition of health been not only the absence of disease but a complete mental, social, and physical well-being (WHOQOL Group, 1995, p. 1404). The short-form version of the World Health Organization’s Quality of Life measurement tools (WHOQOL-BREF) is a 26-item questionnaire that assesses quality of life on physical, psychological, social, and environmental domains (WHOQOL Group, 1998a).

The WHO definition above supports an emerging consensus that QOL is a multidimensional construct conceptualized as separate domains and sub-domains relating to all areas of life (Skevington, 2002; WHOQOL Group, 1995).

In psychology many variables of interest cannot be measured directly, and as *latent constructs* the establishment of their properties remains an ongoing challenge. By using accurate operational definitions a construct’s properties can be evaluated, but reliable and valid measurements can be obtained only when the operational definitions themselves have been rigorously developed (Aiken & Groth-Marnat, 2006). Happiness, subjective well-being, and quality of life are concepts that share common components (Table 1) and arguably, lack standardized operational definitions or criteria. This lack is evident in the interchangeable use of these terms in the research literature (Andrews & McKennell, 1980; Diener et al., 1999; Fordyce, 1986; Shin & Johnson, 1978). The aim of the current study is to clarify relationships between these constructs empirically by applying widely used and well-validated measures of well-being including the OHQ, the World Health Organization Quality of Life Questionnaire (WHOQOL-BREF), the SL Scale, and the Positive and Negative Affect Scale (PANAS).

## Materials and Methods

### Participants

The Auckland University of Technology Ethics Committee granted ethical approved for this study (Ethics Application Number 11/209). New Zealand university students (*n* = 180) recruited in class completed the study questionnaire; from them 35 were males (19.9%), 141 were females (80.1%) and four participants did not provide gender information. We have conducted power analysis to estimate a minimum sample size required for the correlational study with α (two tailed) = 0.05, β = 0.20, and *r* ≥ 0.25, which is *n* = 123 and our sample size is greater. The sample size also satisfied 20 participants per item criteria for principle component analysis with eight study variables (Hair et al., 1995). The participants age ranges from 18 to 55 years, mean age is 24.6 years and standard deviation (SD) is 7.28. About 66 participants (36.7%) identified themselves as New Zealand European, 34 (18.9%) as Asian, 24 (13.7%) as Pasifika, 14 (7.8%) as other European, 7 (3.9%) as Maori, 30 (16.7%) as other ethnicities, and five participants did not indicate their ethnicity. These data has been previously used as a part of psychometric investigation that applied Rasch analysis to evaluate psychometric properties of the OHQ (Medvedev et al., 2016), which is unrelated to the purpose of the current study.

### Instruments

Oxford Happiness Questionnaire (Hills & Argyle, 2002) includes 29 items using six-point Likert scale response format. WHOQOL-BREF quality of life questionnaire (WHOQOL Group, 1998a) includes 26 items with five-point Likert scale response format representing four different domains. The SL scale contains five items presented in seven-point Likert scale format (Diener et al., 1985). The PANAS (Watson, Clark & Tellegen, 1988) includes two subscales measuring positive and negative affect independently. Each scale is composed of 10 adjectives expressing different feelings and emotions like “excited,” “interested” or “distressed” and participants indicate the correspondence of their average feeling to each provided adjective on a five-point Likert scale from “not at all or very slightly” = 1 to “extremely” = 5. The composite subjective well-being scale (SWS) was calculated as a mean of *z*-scores for the SL scale (Diener et al., 1985), the PANAS positive affect subscale and the reversed coded PANAS negative affect subscale (Watson, Clark & Tellegen, 1988). Therefore, the SWS combines positive and negative affect and life satisfaction, which are the main components of subjective well-being suggested by the literature (Table 1).

### Procedure

The study questionnaires were completed by the participants in the lecture theaters of the Auckland University of Technology before lecture. The study complied with local ethical guidelines.

### Data analyses

The data analysis was performed using IBM SPSS program, version 24. The data was screened for normality of distribution and for meeting assumptions for correlation, regression, and principle component analysis. We computed descriptive statistics and examined internal consistency (Cronbach’s alpha and item-to-total correlations) for all included measures with the current dataset. Correlation and regression analyses were conducted to explore the relationships between study variables and the extent to which quality of life domains predict happiness and subjective well-being. Principle component analysis was used to examine communalities and loadings on the first principle component for all study variables.

## Results

### Psychometric properties of the measures

Psychometric properties of the applied scales were tested with our data set. The inter-item total correlation for all the scales were in the permissible range from 0.3 to 0.75 with an exception of the item 2 in OHQ, which correlates with other items at about 0.12. Means, SD, and reliability coefficients for each scale including the OHQ, the WHOQOL-BREF domain scales, the SL and the PANAS positive and negative affect are summarized in Table 2. The majority of the scales have reliability coefficients over 0.8 with the exception of social and environmental domain scales of WHOQOL falling below this number.

**Table 2:** Means, standard deviations, and the Cronbach’s alpha for the Oxford Happiness Questionnaire, the WHOQOL-BREF health, psychological, social, and environmental domains, the Satisfaction with Life, and the PANAS positive and negative affect scales.

	*n*	Number of items	Mean	SD	α_c_
Oxford Happiness Questionnaire	180	29	4.18	0.637	0.903
Physical health (WHOQOL)	179	7	3.78	0.646	0.804
Psychological (WHOQOL)	177	6	3.51	0.660	0.827
Social relationships (WHOQOL)	179	3	3.68	0.815	0.614
Environmental factors (WHOQOL)	179	8	3.70	0.566	0.767
Satisfaction with life	178	5	4.57	1.196	0.871
PANAS positive affect	173	10	3.60	0.615	0.857
PANAS negative affect	172	10	2.32	0.759	0.888

### Correlational analysis

Correlations between the outcome variables, gender, and age are represented in Table 3. The results show that neither gender nor age correlates significantly with any of the scales. The correlations between all well-being related measures are significant and range from moderate to strong.

**Table 3:** Correlation matrix for the outcome well-being variables, gender, and age.

	1	2	3	4	5	6	7	8	9	10	11
1. Gender											
2. Age	0.06										
3. Oxford happiness	−0.09	−0.07									
4. QOL general	0.01	−0.09	0.60**								
5. QOL social	0.05	−0.07	0.51**	0.41**							
6. QOL psychological	−0.08	−0.06	0.83**	0.64**	0.52**						
7. QOL environment	−0.08	−0.06	0.58**	0.48**	0.37**	0.58**					
8. QOL health	−0.06	−0.04	0.69**	0.55**	0.38**	0.68**	0.58**				
9. Life satisfaction	0.01	−0.12	0.72**	0.62**	0.53**	0.76**	0.59**	0.54**			
10. Positive affect	0.01	−0.03	0.77**	0.50**	0.44**	0.77**	0.53**	0.62**	0.65**		
11. Negative affect	0.04	−0.01	−0.61**	−0.52**	−0.36**	−0.59**	−0.43**	−0.62**	−0.50**	−0.45**	
12. Subjective well-being	−0.02	−0.07	0.76**	0.65**	0.52**	0.77**	0.59**	0.66**	0.87**	0.63**	−0.87**

**Notes:**

Oxford happiness is the Oxford Happiness Questionnaire (Hills & Argyle, 2002); QOL is quality of life, QOL general is the general question about quality of life and QOL social, QOL psychological, QOL environment, QOL health are the four domain scales of WHOQOL (WHOQOL Group, 1998a); Life satisfaction is the Satisfaction with Life scale (Diener et al., 1985); Positive affect and Negative affect are PANAS subscales measuring positive and negative affect respectively (Watson, Clark & Tellegen, 1988); Subjective well-being is the composite scale of subjective well-being combining the Satisfaction with Life and the PANAS Positive and reversed Negative affect scales.

** *p* < 0.001.

### Multiple regression analysis

The data satisfied assumptions of multiple regression analysis with skewness and kurtosis values within ±1, no significant outliers, no signs of multicolinearity and variance inflating factor below 5. Multiple linear regression analysis was performed to test regression weights of WHOQOL domains and their significance in predicting happiness as measured by OHQ and subjective well-being measured by the SWS composite measure. Table 4 shows that the WHOQOL domains together explain 73% of happiness on the OHQ. It also shows that the strongest predictor is psychological domain of WHOQOL and environmental factors appear not significant in predicting happiness. All the WHOQOL domains appear significant and together explain about 66% of subjective well-being with psychological domain as the strongest predictor (Table 4).

**Table 4:** Multiple regression results including multiple correlation and standardized Beta coefficients, and *p*-values for the WHOQOL domain scales in predicting happiness and subjective well-being.

	Standardized beta	Sig. (*p*)
**Outcome: happiness** (*R* = 0.85; *R*^2^ = 0.73)		
Physical health (WHOQOL)	0.20	0.000
Psychological domain (WHOQOL)	0.60	0.000
Social relationships (WHOQOL)	0.09	0.046
Environmental factors (WHOQOL)	0.07	0.160
**Outcome: subjective well-being** (*R* = 0.81; *R*^2^ = 0.66)		
Physical health (WHOQOL)	0.21	0.002
Psychological domain (WHOQOL)	0.47	0.000
Social relationships (WHOQOL)	0.13	0.011
Environmental factors (WHOQOL)	0.14	0.020

**Notes:**

Happiness is measured by the Oxford Happiness Questionnaire.

*R*, multiple regression coefficient.

### Principle component analysis

Principle component analysis was first conducted for all applied scales aiming to extract communality of each scale. Extracted communalities and loadings on the single factor for all scales together with total variance explained by scales and total eigenvalue are represented in Table 5. The extracted communalities of the scales range from 0.38 (social relationships) to 0.83 (the OHQ and the psychological domain of WHOQOL).

**Table 5:** Extracted communalities and loadings on the single factor for the Oxford Happiness Questionnaire, the WHOQOL-BREF domains, the Satisfaction with Life, and the PANAS positive and negative affect.

	Number of items	Communalities	Loadings on single component
Psychological (WHOQOL)	6	0.83	0.91
Oxford Happiness Questionnaire	29	0.83	0.90
PANAS positive	10	0.69	0.83
Satisfaction with life	5	0.69	0.83
Physical health (WHOQOL)	7	0.64	0.80
Environmental factors (WHOQOL)	8	0.55	0.74
PANAS negative reversed	10	0.51	0.71
Social relationships (WHOQOL)	3	0.38	0.62

**Figure 1:**
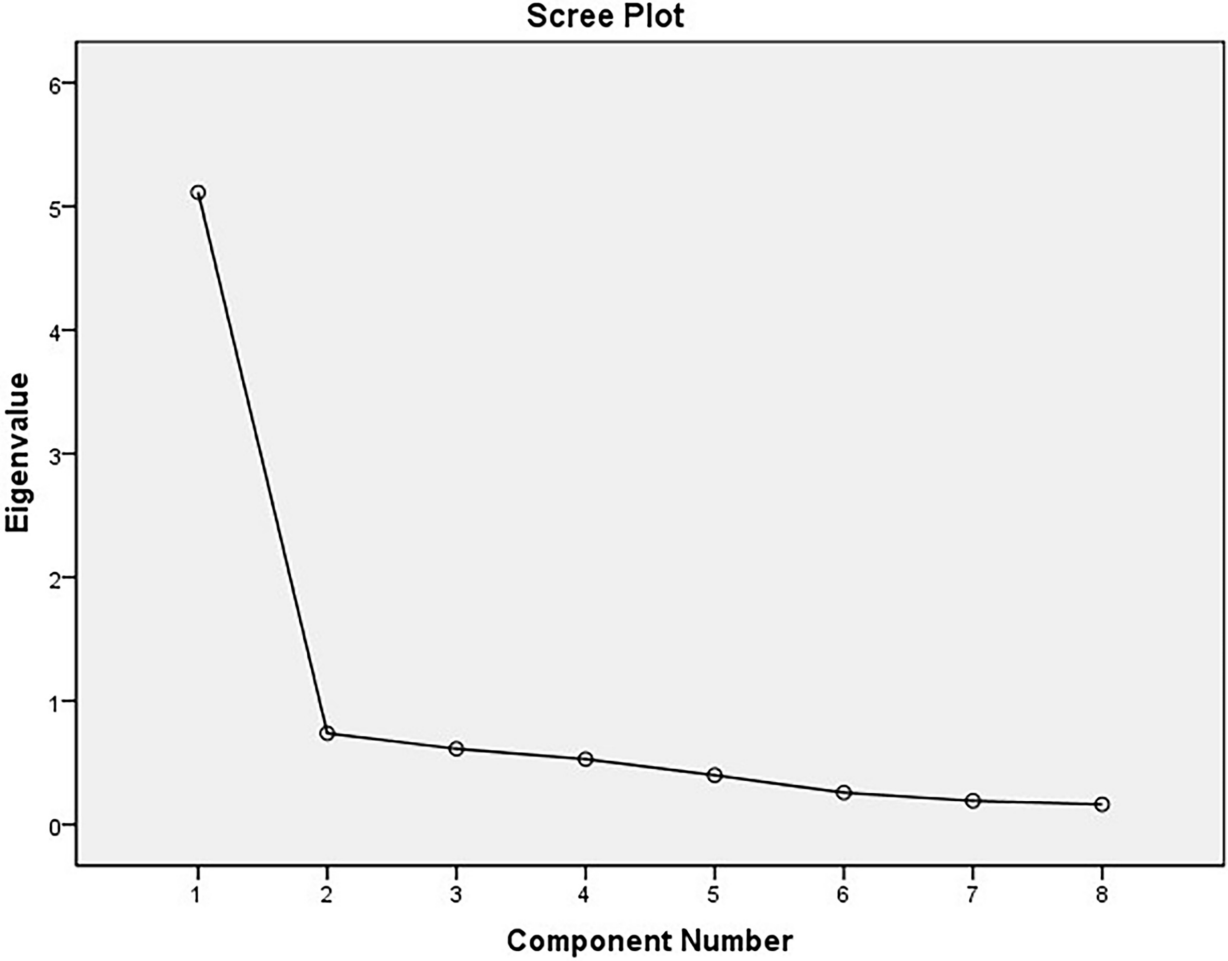
Scree plot represents eigenvalues of extracted components in principal component analysis for the Oxford Happiness Questionnaire, the WHOQOL-BREF domains, the Satisfaction with Life, and the Positive and Negative Affect measures.

Scree plot (Fig. 1) shows the sharp drop and clear Cattell’s cut off point (elbow) after the first principal component and the rest of the plot representing other extracted components is shallow and almost flat.

Alternatively, the SL and the PANAS scales were replaced by the SWS, which followed the same analysis illustrated in Table 6. The extracted communalities range between 0.42 (social relationships) and 0.81 (the OHQ and the psychological domain of WHOQOL).

**Table 6:** Extracted communalities and loadings on the single factor for the Oxford Happiness Questionnaire, the WHOQOL-BREF domains and the Subjective Well-Being composite scale.

	Number of items	Communalities	Loadings on single component
Psychological (WHOQOL)	6	0.81	0.90
Oxford Happiness Questionnaire	29	0.81	0.90
Subjective well-being	25	0.78	0.89
Physical health (WHOQOL)	7	0.67	0.82
Environmental factors (WHOQOL)	8	0.56	0.75
Social relationships (WHOQOL)	3	0.42	0.62

## Discussion

The aim of this study was to investigate the relationship between happiness, subjective well-being, quality of life, and related components by applying widely used scales with satisfactory psychometric properties. Our data offer preliminary clarification of the relationship between happiness, subjective well-being, and quality of life. The results show that all applied well-being measures have high loadings on the global well-being domain that explains about 80% of the variance in the OHQ, the psychological domain of Quality of Life and subjective well-being (Tables 5 and 6). These findings support the proposed global dimension of well-being that transcends relative distinctions between specific components contributing to the overall wellness (Kashdan, Biswas-Diener & King, 2008; Hills & Argyle, 1998, 2002; Joseph & Lewis, 1998). These results also provide support for interchangeable use of happiness and subjective well-being, and suggest that these constructs and quality of life domains may be considered as facets of the global well-being construct.

Widely used well-being measures capture subjective evaluation of individual’s condition and top-down approach suggests that people are happier if they evaluate their life including its eudemonic aspects in a positive way (Diener et al., 1999; Diener, 1984). In contrast, negative evaluations diminish well-being, may discount eudemonic components and lead to psychological conditions such as depression or anxiety (Diener et al., 1999; Beck, 1991, 1993). Therefore, moderate to strong correlations found between subjective well-being measures in this study were expected according to this approach. The strongest relationship is evident between the OHQ, psychological domain of the WHOQOL, the SL, the PANAS Positive affect, and the SWS (Table 3). It is likely that correlation values were suppressed due to unrepresentative student sample with substantial proportion of international students for whom English is a second language, which might have produced a response bias. Also, the correlation values could suffer from inconsistent item wording in different scales. For example, the WHOQOL items ask the participants to evaluate their affective experiences for the last two weeks (WHOQOL Group, 1998a), whereas the PANAS measures how the participants feel on average (Watson, Clark & Tellegen, 1988). Thus, the correlations could be higher if uniform wording was used for all scales and applied to a larger sample more representative of the general population.

Furthermore, the results show that the psychological, physical health, social, and environmental domains of WHOQOL together explain 73% of happiness measured by the OHQ and 66% of subjective well-being. In both cases psychological domain was the strongest predictor, but environmental factors explain only 14% of the variance in subjective well-being and were found not significant in predicting happiness. These data suggest that environment does not appear as relevant determinant of individual happiness. However, psychological domain appeared as strongest predictor of both happiness and subjective well-being in contrast to both environment and social relationships. The social relationships explain the least amount of variance in the global well-being construct indicating that they may play important but not the major role in individual’s well-being of the current sample. These results are consistent with earlier studies supporting top-down approach and emphasizing the role of individual’s cognition in subjective happiness (Andrews & McKennell, 1980; Andrews & Withey, 1976; Butler et al., 2006).

The main problem to address redundancy issue is the proposed multidimensional structure of WHOQOL in which the four domains are typically assessed independently without providing a combined quality of life score (WHOQOL Group, 1998b). However, our results suggest that the psychological domain of WHOQOL can be used as an alternative brief measure of happiness or subjective well-being, which is an advantage, because it is a six-item scale with good reliability compared to the 29-item OHQ.

Tested with our data set, satisfactory psychometric properties of all scales used in the study appeared consistent with earlier research (Hills & Argyle, 2002; Diener et al., 1985; Watson, Clark & Tellegen, 1988; WHOQOL Group, 1998a) with the exception of item 2 in the OHQ, which correlates with other items at 0.12, below commonly acceptable level of 0.3. Thus, discarding this item would slightly increase reliability of the OHQ, which is recommended for future application of this scale. However, it is unlikely that this item could have strong influence on overall sufficiently high reliability of the scale (α = 0.90).

## Limitations

The common limitations of subjective well-being research refer to participants’ transient mood states and other contextual influences, which might affect participants’ responses (Eid & Larsen, 2008). However, these effects were minimized because the data were collected in different classes. The other limitation of this study refers to the modest sample size and disproportionally larger number of female participants (80.1%) comparing to male (19.9%), which limits generalization of the findings to the male population. In this study we used all measures in their original form without enhancement of their psychometric properties to maintain consistency with studies conducted earlier. Recently proposed modification of happiness and quality of life assessment tools (Medvedev et al., 2016; Krägeloh et al., 2016) may contribute to more accurate estimations of relationships between these happiness and well-been measures. However, this would require similar psychometric enhancements of all other measures (e.g., PANAS) involved in the analyses, which are not available to date.

## Future Directions

Further research should investigate the relationship between happiness, subjective well-being, and quality of life among more diverse populations, including people differing in socio-economic status and health conditions, as these dimensions have proved to be crucial in the assessment of well-being under its different formalizations. Finally, the research should focus on development of more accurate instruments for assessment of happiness and subjective well-being by considering the WHOQOL domains and other relevant measures.

## Conclusions

Taken together, the findings of this study provide support for a global well-being dimension and interchangeable use of terms happiness, subjective well-being, and psychological quality of life with the current sample and measures. The WHOQOL measures happiness or subjective well-being by its psychological domain but in addition includes subscales focused on measurement of perceived physical health and more externally oriented domains such as social relationships and environmental factors. These differences should be considered in measurement definitions to refine reliability and validity. The findings of this study contribute to better understanding of the relationships between happiness, subjective well-being, and quality of life, which is necessary for more accurate assessment of these constructs. Also, these findings have implications for the enhancement of people’s well-being, happiness, and quality of life through development of contentment and emotional stability. Further investigation with larger heterogeneous samples and other well-being measures is warranted.

## Supplemental Information

10.7717/peerj.4903/supp-1 Supplemental Information 1 Well-being measures raw data file (n = 180).

## Additional Information and Declarations

### Data Availability

The following information was supplied regarding data availability: The raw data are provided in the Supplemental File.
